# Supplementary material for: Ex Vivo Fluorescence Confocal Microscopy (FCM) Ensures Representative Tissue in Prostate Cancer Biobanking: A Feasibility Study
Source: Int J Mol Sci. 2022 Oct 11;23(20):12103. doi: 10.3390/ijms232012103 (PMC9603154; doi:10.3390/ijms232012103)
Supplement: Supplementary file 1 [file ijms-23-12103-s001.zip › ijms-1911311-supplementary.pdf]

Supplementary Materials

Ex Vivo Fluorescence Confocal Microscopy (FCM) Ensures Representative Tissue in Prostate Cancer Biobanking: A Feasibility Study

Ulf Titze [1,*], Johannes Sommerkamp [1], Clara Stege [1], Fried Schneider [2], Christoph Brochhausen [3,4], Birte Schulz [1], Barbara Titze [1], Furat Abd Ali [2], Sasa Pokupic [2], Karl-Dietrich Sievert [2,†] and Torsten Hansen [1,†]

[1] Institute of Pathology, University Hospital OWL of the University of Bielefeld, Campus Lippe, 32756 Detmold, Germany
[2] Department of Urology, University Hospital OWL of the University of Bielefeld, Campus Lippe, 32756 Detmold, Germany
[3] Institute of Pathology, University of Regensburg, 93053 Regensburg, Germany
[4] Central Biobank Regensburg, University Hospital Regensburg, 93053 Regensburg, Germany
* Correspondence: ulf.titze@klinikum-lippe.de (U.T.); Tel.: +49-05231-72-3451
† K.-D.S. and T.H. equally contributed to this work.

Content:

Figure S1: Study design
Figure S2: Analysis of biobank samples
Table S1: Processing Times of FCM-Analyses
Table S2: DNA extraction from representative biobank samples of tumorous and normal tissue

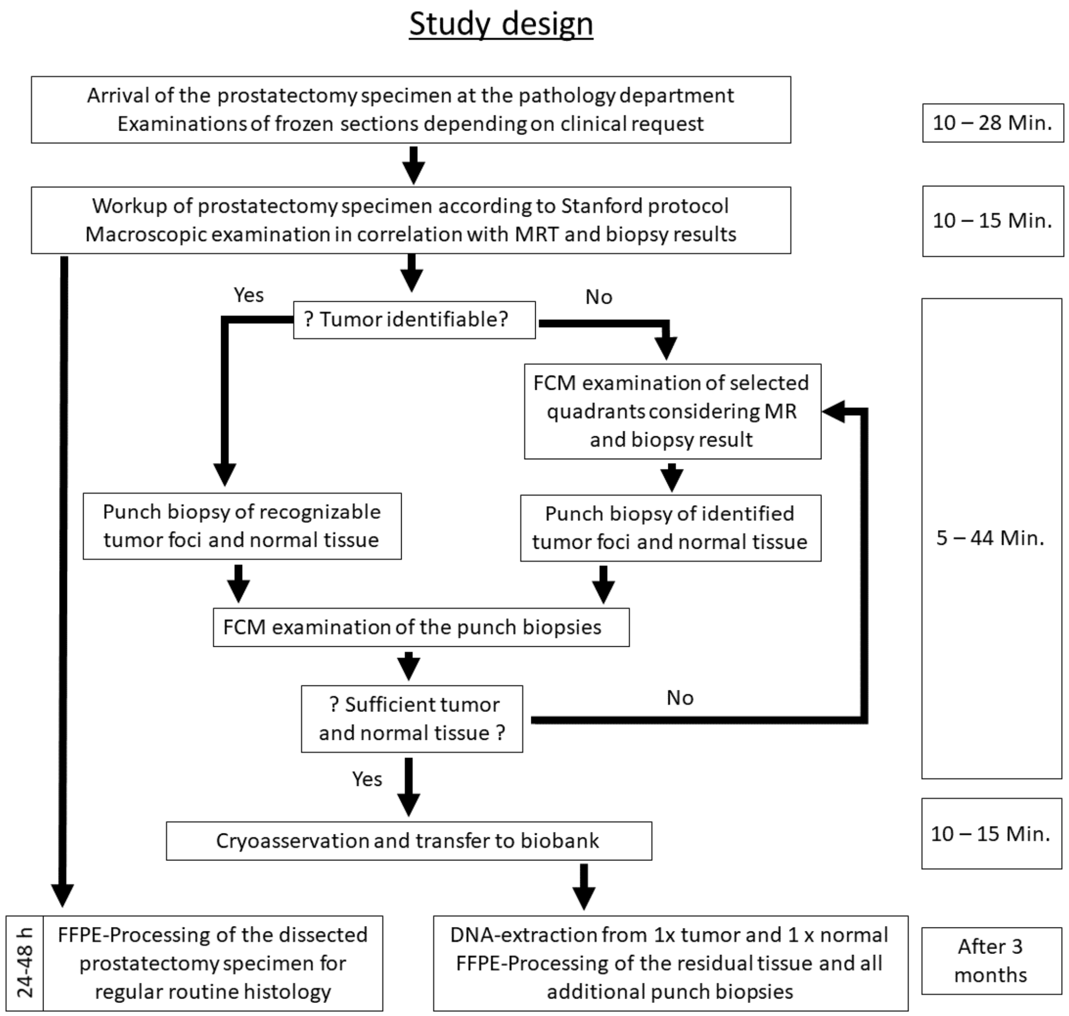

Figure S1: Workflow of surgical dissection, macroscopic examinations and FCM analyses of prostatectomy specimens and biobank samples.

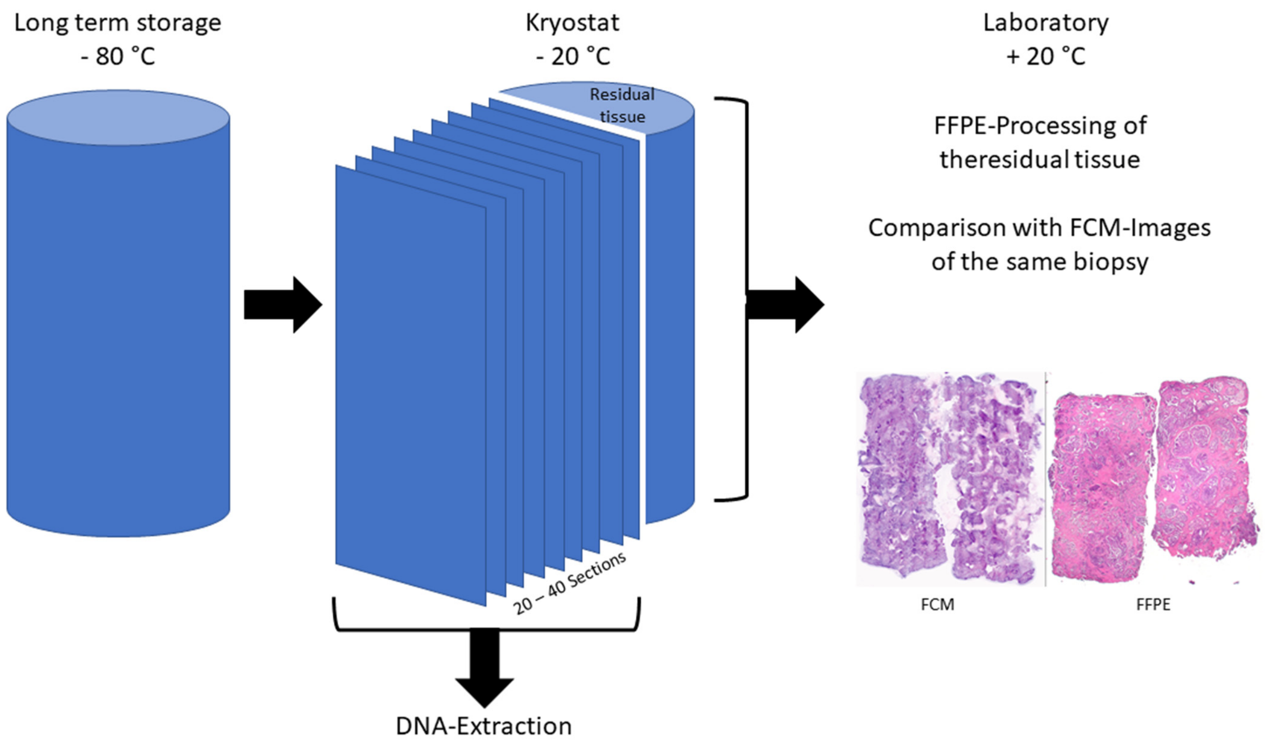

Figure S2: Analysis of biobank samples
The selected biopsy core is taken from the biobank (-80 °C) and brought to cutting temperature (-20 °C) in the cryostat. Depending on the size, 20-40 cryostat sections are prepared from the biopsy for DNA extraction. The residual tissue remaining on the stamp is further processed according to FFPE procedure. The HE-stained sections are compared with the FCM scans of the same biopsy.

Table S1: Total Processing times of FCM-Analyses and resulting ischemia time of the biobank samples

	Number of FCM-Scans*			Duration of FCM-	Ischemia
Pat	Small Biopsies	Whole Quadrants	Total	Examinations [min]	[min]
P01	3	1	4	16	n.a.
P02	2	0	2	24	38
P03	2	0	2	6	40
P04	3	0	3	9	n.a.
P05	3	0	3	10	27
P06	2	0	2	6	n.a.
P07	2	0	2	8	40
P08	2	0	2	5	20
P09	2	0	2	8	23
P10	3	0	3	9	49
P11	3	0	3	33	41
P12	5	0	5	17	39
P13	1	0	1	5	90
P14	3	0	3	18	56
P15	2	0	2	9	41
P16	2	0	2	6	55
P17	3	0	3	13	51
P18	2	2	4	24	43
P19	2	2	4	16	49
P20	4	1	5	37	76
P21	1	0	1	5	36
P22	5	1	6	31	83
P23	2	1	3	35	73
P24	6	0	6	27	78
P25	2	0	2	11	56
P26	3	0	3	16	39
P27	2	1	3	18	49
P28	3	0	3	12	32
P29	1	0	1	5	59
P30	5	1	6	44	52
P31	2	0	2	6	26
P32	2	0	2	6	24
P33	4	0	4	14	34
P34	2	0	2	5	75
P35	4	0	4	30	109
P36	3	0	3	15	79
P37	2	0	2	7	22
P38	3	0	3	8	23
P39	3	0	3	20	48
P40	5	0	5	28	56
Ø			3.0	15.6	49.5
±			1.3	10.5	21.3
Max			6	44	109
Min			1	5	20

Processing times were reconstructed based on the documented times of specimen receipt and frozen section reporting, as well as the metadata of the FCM images. For the punch biopsy* scans, in some cases (especially P13, P21, P29) multiple samples from different collecting sites were examined in one single scan, resulting in a significant reduction of processing time.

Table S2: DNA extraction from representative biobank samples of tumorous and normal tissue.

**Pat.**	**Tumor tissue**						**Normal tissue**			
	**Block**	**PCa [%]**	**ISUP**	**DNA [ng/µl]**	**$A_{260/280}$**	**$A_{260/230}$**	**Block**	**DNA [ng/µl]**	**$A_{260/280}$**	**$A_{260/230}$**
P01	RE1	30	4	72.1	1.86	2.51	LD2	12.6	1.79	2.46
P02	LB1	100	3	66.3	1.99	2.20	RF2	45.8	1.91	2.20
P04	RD2	20	1	140	1.91	2.16	LD2	580	1.92	2.27
P05	LC2	70	2	498	1.94	2.23	RC2	230	1.94	2.35
P06	RC2	80	3	241	1.91	2.17	LB2	163	1.93	2.31
P07	RD2	80	2	431	1.93	2.17	LE2	51.2	1.83	2.10
P08	LD2	100	3	370	1.96	2.28	RD2	570	1.96	2.23
P09	RD2	100	1	257	1.90	2.26	LC1	35.8	1.84	1.93
P10	RD1	90	1	125	1.92	2.04	RD2	450	1.91	2.28
P11	LB2	100	2	228	1.87	1.84	RD2	243	1.88	2.10
P12	LC2	100	2	257	1.96	2.35	LII2	108	1.97	2.20
P13	LC2	90	2	25.6	1.97	2.27	RE2	106	1.89	2.22
P14	RC2	100	3	560	1.94	2.34	LC1	298	1.90	2.34
P15	RB2	100	3	459	1.95	2.28	LE2	225	1.85	2.22
P16	LE2	50	2	70.7	1.99	2.07	RC2	393	1.92	2.24
P17	RD2	100	1	310	1.92	2.25	LE2	177	1.97	2.06
P19	LE2	100	3	76	1.96	2.20	RD1	297	1.93	2.30
P21	RD2	70	1	203	1.89	2.27	LD1	99.9	1.91	2.37
P22	RC2	10	1	258	1.95	2.24	RE2	228	1.86	2.15
P24	LE1	75	1	368	1.93	2.33	LE2	306	1.94	2.25
P25	LB1	70	1	339	1.92	2.34	RB2	570	1.92	2.20
P26	LD1	40	1	119	1.86	2.10	LB1	303	1.90	2.31
P27	LC2	70	5	530	1.90	2.21	RD2	570	1.87	2.16
P28	RB2	40	2	257	1.93	2.33	RF1	114	1.92	2.20
P29	LB2	100	2	520	1.92	2.32	RB1	299	1.95	2.12
P30	RB2	20	2	386	1.95	2.27	LD1	492	1.90	2.20
P31	RB2	70	2	362	1.97	2.19	LE1	510	1.94	2.31
P32	RE2	100	3	590	1.95	2.31	LE2	264	1.96	2.23
P33	LII1	90	2	405	1.95	2.23	RD2	425	1.93	2.23
P34	LC1	80	2	239	1.95	2.26	RC2	365	1.95	2.11
P35	LD2	80	3	342	1.94	2.20	RC2	116	1.92	2.25
P36	RC2	100	2	449	1.95	2.25	LE1	461	1.93	2.31
P37	RC2	90	1	384	1.96	2.22	LB2	208	1.91	2.38
P38	RC2	100	4	530	1.90	2.21	LC1	211	1.97	2.28
P39	RC1	100	1	189	1.98	2.31	LB1	227	1.97	2.24
P40	RB1	50	4	142	1.96	2.19	RD1	570	1.93	2.03
Ø		76.67		300.0	1.93	2.23		286.8	1.91	2.22
±		27.49		159.6	0.03	0.11		174.0	0.04	0.11
Max		100		590.0	1.99	2.51		580.0	1.97	2.46
Min		10		25.6	1.86	1.84		12.6	1.79	1.93

Representative samples of tumor and normal tissue were obtained in 36/40 (90%) patients. Sufficient amounts of DNA with appropriate purity for further molecular analyses could be extracted from the samples.
